# TM-WSNet: A precise segmentation method for individual rubber trees based on UAV LiDAR point cloud

**DOI:** 10.1016/j.plaphe.2025.100093

**Published:** 2025-08-21

**Authors:** Lele Yan, Guoxiong Zhou, Miying Yan, Xiangjun Wang

**Affiliations:** a Central South University of Forestry and Technology, Changsha, 410004, China; b Rubber Research Institute of the Chinese Academy of Tropical Agricultural Sciences, Haikou, Hainan, 571101, China

**Keywords:** Rubber tree segmentation, Hybrid feature extraction module, Wavelet grid sampling, Multi-level feature fusion, Scale optimization algorithm

## Abstract

Rubber products have become an important strategic resource in the global economy. However, individual rubber tree segmentation in plantation environments remains challenging due to canopy background interference and significant morphological variations among trees. To address these issues, we propose a high-precision segmentation network,TM-WSNet (Spatial Geometry Enhanced Hybrid Feature Extraction Module–Wavelet Grid Feature Fusion Encoder Segmentation Network). First, we introduce SGTramba, a hybrid feature extraction module combining Grouped Transformer and Mamba architectures, designed to reduce confusion between tree crown boundaries and surrounding vegetation or background elements. Second, we propose the WGMS encoder, which enhances structural feature recognition by applying wavelet-based spatial grid downsampling and multiscale feature fusion, effectively handling variations in canopy shape and tree height. Third, a scale optimization algorithm (SCPO) is developed to adaptively search for the optimal learning rate, addressing uneven learning across different resolution scales. We evaluate TM-WSNet on a self-constructed dataset (RubberTree) and two public datasets (ShapeNetPart and ForestSemantic), where it consistently achieves high segmentation accuracy and robustness. In practical field tests, our method accurately predicts key rubber tree parameters—height, crown width, and diameter at breast height with coefficients of determination (R^2^) of 1.00, 0.99, and 0.89, respectively. These results demonstrate TM-WSNet's strong potential for supporting precision rubber yield estimation and health monitoring in complex plantation environments.

## 1 Introduction

As a key economic crop widely planted in tropical areas, the natural rubber produced by rubber trees plays an irreplaceable role in industry and daily life. Accurate segmentation and identification of individual rubber trees is essential for obtaining key growth parameters such as crown diameter, diameter at breast height and growth status during the growth cycle of rubber trees [1]. These parameters have significant implications for monitoring the growth of rubber trees, evaluating their health, and guiding scientific planting management [2]. Therefore, a high-precision single rubber tree segmentation technology can not only improve the scientificity and accuracy of rubber tree planting, but also promote technological innovation and feasibility of the rubber industry.

Three-dimensional point clouds with geometric structure information provide important ideas for researchers to solve segmentation problems [3]. In the processing of 3D point cloud, the traditional machine learning method plays an important role. In the processing of three-dimensional point clouds, machine learning plays an important role. For example, Stefano Puliti [4] and others constructed laser scanning data sets in different areas, providing important data support for learning to segment single trees. Wang [5] proposed a coupled algorithm framework to effectively estimate the coronal volume of point clouds and evaluate forest biomass. Hakula [6] and others used the Fuzzy k-Nearest Neighbors classifier to segment laser point cloud data into individual trees, providing an important reference for assessing forest biomass. Li et al. [7] used an improved DBSCAN to detect tree trunks and initial clustering results, and used the Hough circle fitting method to modify the trunk detection results. Finally, individual tree segmentation was achieved through layer-by-layer clustering based on region growth. Although these can effectively separate neighboring trees, the additional data structures used will result in large computational and memory costs [8]. Moreover, the single tree segmentation effect for bifurcated trees, multi-stem trees and other complex trees is relatively poor, and the rubber tree data set is composed of these complex tree shapes. Therefore, machine learning cannot handle high-precision single tree segmentation, still faces huge challenges.

In recent years, with the development of deep learning technology, 3D point cloud data processing through depth models has gradually become a mainstream trend. Maciej et al. [9] constructed a CNN-based SegmentAnyTree model for tree segmentation on multiple laser point cloud datasets, and achieved high performance. Xia et al. [10] proposed an innovation combining deep semantic segmentation and clustering, first using RandLA-Net-based deep semantic segmentation network point cloud data for segmentation, and then using clustering to extract point cloud data of a single tree from it. Experimental results show that the proposed method performs well in complex scenes and significantly improves the segmentation accuracy of three-dimensional trees. However, these rely only on local dependencies while ignoring global features, resulting in limited segmentation performance.

Recently, with the deep learning, Transformer-based model framework, with its unique self-attention mechanism, it has shown excellent potential in point cloud context modeling. For example, the Point Transformer proposed by Zhao et al. [11] effectively captures remote dependencies in point clouds by introducing a self-attention mechanism, thereby improving segmentation accuracy [12]. Point Cloud Transformer (PCT), a new framework proposed by Guo et al. [13], enhances input through farthest point sampling and nearest neighbor search, and has powerful feature extraction capabilities. In addition, Wang et al. [14] designed a Transformer architecture based on global perception and local structure fitting to learn detailed point cloud shape information and enhance the perception ability of point cloud features. In addition, inspired by the structured state space model (S4) [15], the global perception capability shown by the newly proposed Mamba [16] has attracted widespread attention from researchers, and compared with Transformer's *O*(*n*^2^)complexity, Mamba's computational complexity is *O*(*n*), which makes it widely tried in two-dimensional images [17,18]. Therefore, inspired by Mamba in 2D images, Liang et al. [19] recently proposed to combine point cloud grouping with Mamba encoder to build PointMamba network, while PC-Mamba [20] built by Zhang et al. combines Mamba with PointMLP to fully explore the potential of Mamba in point cloud.

The above research provides valuable insights for the realization of single-tree segmentation of rubber trees. However, our research has identified three major challenges that still need to be addressed for accurate segmentation of individual rubber trees:

1. Rubber tree canopy boundary blurriness. Rubber trees are typically planted densely, making the boundaries of their canopies prone to confusion with adjacent tree canopy points and other non-target elements. Additionally, near-ground background noise points and trunk points may be difficult to separate due to their similar density, making it challenging to clearly delineate the boundaries of individual trees.
2. Irregular rubber tree structure. There are significant morphological differences among individual rubber trees, including variations in trunk height, crown shape, and leaf distribution. These irregularities add complexity to the segmentation of individual trees, especially in high-density plantations, where the irregular structural features of trees are often difficult to accurately identify and separate.
3. The setting of the learning rate. The learning rate has different effects on model segmentation at various scale resolutions. If the learning rate is not set properly, it may cause the model to learn large-scale features (such as the tree canopy) and small-scale features (such as the tree trunk) unevenly. This imbalance can prevent the model from accurately segmenting trees at different scales, thereby affecting overall segmentation accuracy.

To address the challenge of fuzzy canopy boundaries in rubber tree samples, Hu et al. [21] introduced a novel local feature aggregation module. This approach effectively preserves local geometric details and enhances the representation of local features by progressively perceiving the point cloud field. However, despite using random sampling to improve efficiency, this strategy may lose key geometric feature points, especially at locations with significant structural changes or object boundaries. Furthermore, Yang et al. [22] proposed a novel Gumbel subset sampling point cloud modeling method, which uses hierarchical selection of local key features and replaces multi-head attention with the lightweight Group Shuffle Attention (GSA), effectively improving segmentation accuracy. However, the lightweight design may result in insufficient global modeling capability, particularly when handling rubber tree samples with significant structural differences. Additionally, Liang et al. [19] achieved superior performance on multiple datasets using a simple non-hierarchical Mamba encoder as the backbone, providing a foundation for the use of Mamba in 3D point clouds. Meanwhile, Wang et al. [23] proposed the PoinTramba hybrid framework, leveraging the combined advantages of Transformer and Mamba. This method was primarily tested on standard scene datasets such as ScanObjectNN, ModelNet40, and ShapeNetPart, which differ significantly from the dense planting, high overlap, and fuzzy boundaries of rubber tree environments. Therefore, there is a need to develop an efficient feature extraction module to enhance the model's ability to model irregular structures and fuzzy boundaries, and accurately capture key geometric features in complex point clouds.

To effectively address the issue of irregular rubber tree structures, sampling has gained widespread attention, with Farthest Point Sampling (FPS) being of particular interest to researchers due to its robustness [24,25]. It is primarily used in the downsampling process [26,27]. FPS is also employed in large-scale point cloud models that require high computational efficiency, such as PointNet++ [28] and voxel networks [29]. Additionally, Wang and Zhai et al. [30] proposed a multi-scale fusion module that adaptively processes information at different scales, enriching global information and effectively enhancing the global perception of rubber tree structures. However, in rubber tree segmentation, irregular structures are not only reflected in geometric shapes but also in spatial hierarchical features. The MF module lacks explicit modeling of spatial structures. Zhang et al. [31] proposed a novel masked autoencoder that adopts a multi-scale masking strategy to progressively capture fine-grained features of 3D shapes, thereby enhancing the model's generalization capability. Hui et al. [32] constructed a pyramid VLAD module that aggregates multi-scale feature maps from different point clouds into a global descriptor, achieving remarkable segmentation performance across diverse point cloud datasets. However, the pyramid VLAD module, while effective for global multi-scale feature construction, exhibits limited discriminative power when applied to rubber tree samples requiring fine-grained segmentation. Therefore, designing a feature extraction framework that balances local structural detail and spatial modeling is crucial for improving the recognition of complex individual structures in rubber trees.

To effectively address the problem of optimal learning rate setting, Loshchilov and Hutter [33] proposed a learning rate strategy with a periodic restart mechanism. By cyclically adjusting the learning rate and restarting the optimization process, their method helps the model escape from local optima. However, this approach suffers from difficulties in jointly coordinating the learning intensity across multi-scale branches, which may lead to imbalanced gradient propagation between different scales. He et al. [34] introduced a learning rate scheduling framework integrated with the RAdam optimizer, which ensures performance balance in feature learning across different scales by dynamically adjusting the learning rate and optimizer parameters. Smith et al. [35] proposed a cyclical learning rate (CLR) strategy that enables rapid convergence during the early stages of training and stabilizes the model in favorable weight regions later on, thus effectively balancing the learning of both coarse and fine-scale features. However, due to the highly irregular structure and complex spatial hierarchy of rubber trees, their segmentation tasks rely more heavily on meticulous feature extraction and staged optimization. The existing CLR strategies adopt a unified periodic scheduling approach, which fails to fully accommodate the diverse learning rate requirements across different feature scales during training. Therefore, it is essential to design a scale-aware learning rate scheduling mechanism to enhance the robustness and accuracy of segmentation in irregular structural scenarios.

Therefore, in response to the above problems, the contributions of this paper are as follows:

1. To resolve the challenge of unclear rubber tree boundaries: We propose a hybrid feature extraction module that groups the point cloud data, using a geometry-enhanced grouping Transformer to extract local features within each group, and Mamba to capture global dependencies between different group sequences. This module effectively captures the regional spatial geometric features of the point cloud, thereby improving the model's ability to capture boundary and structural information.
2. To effectively handle the morphological complexity between individual rubber trees. We propose a wavelet mesh feature fusion encoder that performs initial feature sampling using wavelet meshes, and employs a hierarchical progressive weighted fusion feature extraction method to capture multi-scale features. This improves the model's ability to capture global spatial information and the complex shape characteristics of rubber trees.
3. To address the issue of uneven learning across different scales in networks. We design a scale optimization algorithm (SCPO). This algorithm effectively controls the search boundaries using the mathematical properties of the hyperbolic sine and cosine functions and combines the particle swarm optimization (PSO) algorithm's efficient information exchange and position update mechanisms to balance the local and global optimal solutions. It significantly improves the search capability and convergence speed of the algorithm, enhancing the model's learning ability at different scales.

## 2 Materials and methods

### 2.1 Study area

Hainan Island (19°20′N-20°10′ N, 108°21′ E−111°03′ E) is the second largest island in China with an area of approximately 4.3 million hectares. Hainan Island is located in the leeward zone area, and the climate conditions are warm and humid, which is very suitable for tropical agriculture [36]. The land on the island is mainly used for forest and agricultural cultivation, of which rubber tree plantations account for more than a quarter of the total forest area. The research area of the Rubber Research Institute of the Chinese Academy of Tropical Agricultural Sciences is located in Danzhou City. This area provides rich rubber tree resources and provides a natural experiment for studying the growth characteristics and ecological conditions of rubber trees.

### 2.2 Data acquisition

The experimental data were collected on March 5, 2023 ​at the Rubber Research Institute of the Chinese Academy of Tropical Agricultural Sciences, Danzhou City, Hainan Province. The flight altitude of the UAV was set at 80 ​m, 50–60 ​m from the canopy, and the average flight speed was 6 ​m/s. To ensure coverage completeness, the lane spacing was set at 10 ​m, and the lateral overlap rate was about 65 ​% to improve the density and accuracy of the point cloud data. In the data acquisition process, we use DJI M300 RTK drone produced by Shenzhen DJI Innovation Technology Co., Ltd, equipped with CBI Lite laser radar measurement system of Chengdu Aolunda Technology Co., Ltd, for data acquisition, the specific parameters of the radar measurement system are as follows: wavelength of 905 ​nm, emission frequency of 10 ​Hz, maximum point rate of 1.92 million, the scanning range is 360°, absolute relative accuracy is 5 ​cm, and the maximum detection distance is 120 ​m.

### 2.3 Data preprocessing

In this paper, a high-resolution three-dimensional model of rubber plantations is generated by using laser radar to collect three-dimensional point cloud data of rubber plantations in Hainan Province.

**Fig. 1:**
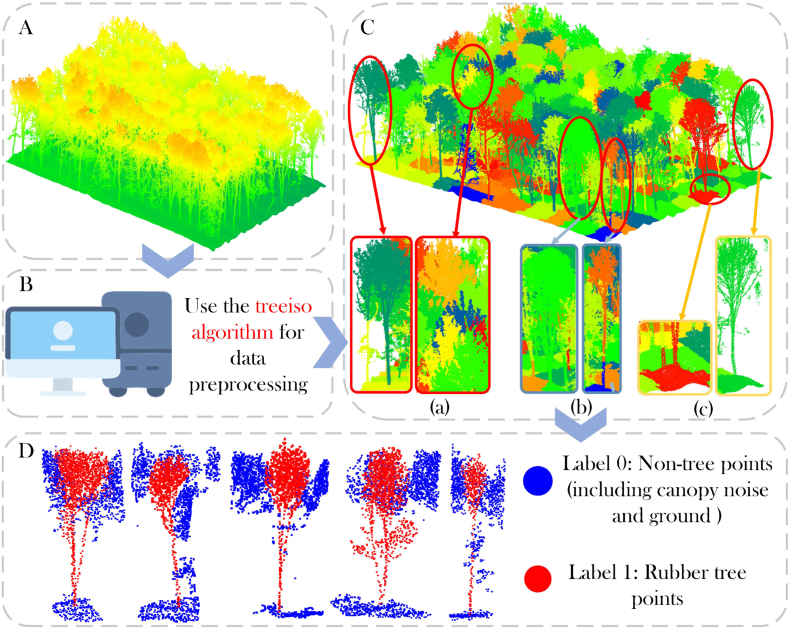
Rubber tree dataset processing workflow.

In the initial data processing stage, we employed the 3D Graph-Based Individual-Tree Isolation(treeiso) [37] algorithm to perform a rough pre-segmentation of the block-based forest point cloud data. The task of the treeiso algorithm at this stage was to delineate the approximate spatial range of each rubber tree and extract the main structure point cloud of the tree samples. However, due to the morphological differences between trees and the blurry boundary issues of rubber trees, errors inevitably occur during the coarse segmentation process. When there is crown overlap between adjacent rubber trees, the treeiso algorithm tends to over-segment, misclassifying part of the crown points of other tree samples as part of the current tree sample, thereby generating crown noise points. For example, in the visualization results on the left side of Fig. 1C.(a), the crown points of an adjacent yellow rubber tree are mistakenly classified as part of the green sample's crown. Additionally, due to the uneven topography of the forest, the treeiso algorithm struggles to accurately remove ground point clouds. For instance, in the visualization results on the left side of Fig. 1C.(c), the near-ground sample of the red rubber tree mistakenly includes key root and trunk points from the adjacent yellow rubber tree. To avoid affecting the integrity of the sample features, we chose to retain the ground point cloud and categorize it along with the crown noise points.

After the preliminary data processing, we selected rough segmentation samples containing complete individual rubber trees and imported them into the 3D point cloud processing software CloudCompare for high-precision manual registration. To minimize the error accumulation caused by early preprocessing, we labeled the main points of the target rubber tree as “tree” and all points from non-target rubber trees as “other” in the software. The “tree” class was labeled as 1 and the “other” class as 0. This labeling provides a unified training semantic foundation for the subsequent fine segmentation model based on the TM-WSNet network.

In order to further enrich the geometric feature information of the rubber tree point cloud data, we computed the normal vectors of all points using the Principal Component Analysis (PCA) method provided by CloudCompare. This step allows each point to be enriched with surface orientation information, enabling segmentation based not only on spatial position but also on normal vector attributes. The segmented point cloud data is then exported to a. txt format for further processing.

Subsequently, CloudCompare was used to segment a large amount of rubber tree point cloud data and obtain thousands of independent rubber tree samples. To ensure the diversity and complexity of the data, we selected 1334 rubber trees at different growth stages and under different conditions for a detailed analysis and finally selected 600 rubber trees for experiments. This sample number not only ensures the representativeness of the data and the feasibility of the experiment, but also facilitates subsequent resource management and data. Finally, the data set was randomly divided into a training set and a test set at a ratio of 4:1, resulting in a training set containing 480 rubber tree point clouds and a test set containing 120 rubber tree point clouds. This data division not only ensures data coverage, but also provides sufficient data support for model training and evaluation.

### 2.4 TM-WSNet

we design a TM-WSNet model with a typical encoder-decoder backbone architecture. In the point cloud initialization stage, the raw point cloud data is voxelized into sparse voxels, where each voxel aggregates features from the contained points. These initial features are then mapped into a high-dimensional embedding space using the patch embedding module, while retaining the same sparse point cloud format as the input. This provides the encoder with high-dimensional, spatially sparse input data. In the up-sampling part, the point cloud data are enhanced by wavelet features, and then divided into fixed-size voxel meshes, and the point features in the meshes are maximally pooled. In the feature extraction stage, we extract point cloud features through the Spatial Geometry Enhanced Transformer-Mamba Feature Extraction Module(SGTramba), and then perform multi-level feature weighted fusion and jump connection on the processed point cloud features. In the decoder stage, we use the mapping de-pooling operation to decode the features, gradually restore the high-resolution features of the point cloud, and improve the accuracy of the decoding process. After decoding, the segmentation head module classifies the high-resolution features output by the decoder, outputs the class probability distribution of each point, and generates the segmentation label of each point. In addition, during the training period, we use a self-designed scale optimization algorithm to search for the optimal learning rate to improve the segmentation accuracy.

#### 2.4.1 Spatial Geometry Enhanced Transformer-Mamba Feature Extraction Module(SGTramba)

**Fig. 2:**
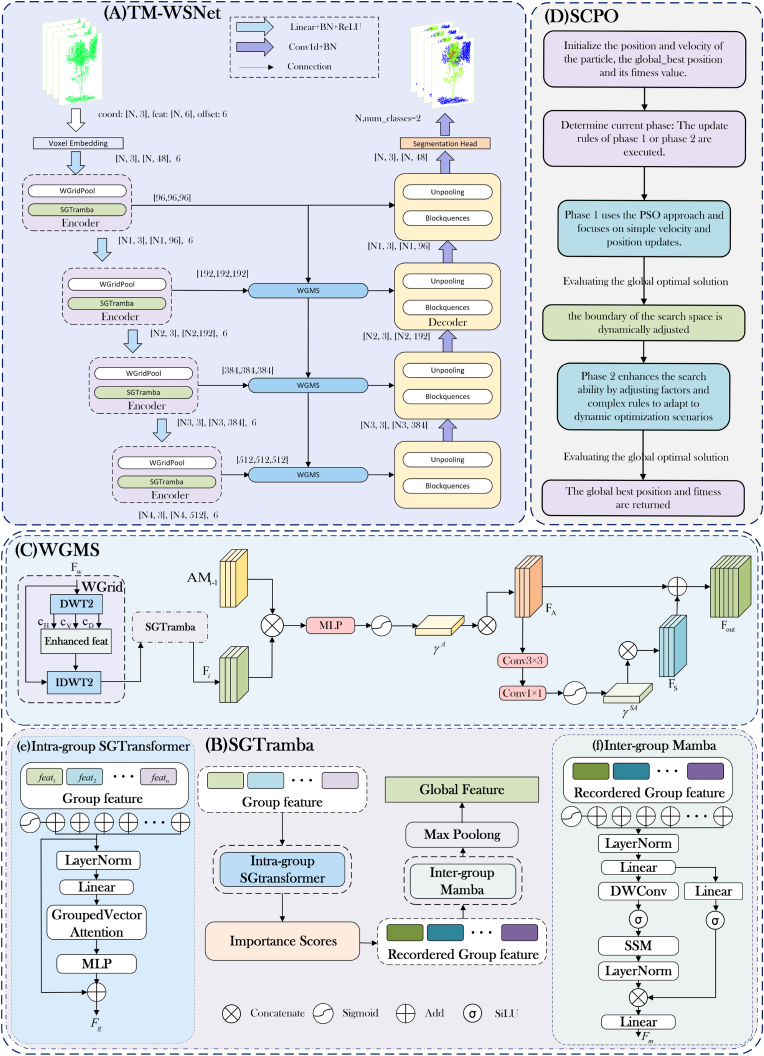
The structure of the TM-WSNet.

How to deal with the non-uniform geometric position information in complex scenes is an indispensable factor to enhance the segmentation accuracy of the rubber tree point cloud segmentation model. To solve this problem, we propose a hybrid feature extraction module (SGTramba) that fuses Transformer and Mamba. First, for the input features, we divide them into *G* groups. In each group, we use the intra-group module composed of the group transformer to generate the intra-group feature ${f_{g}}^{G}$, where *g* represents the *g* first group. Then calculate the importance scores between different groups and sort them in descending and ascending order to get. After the intra-group feature reordering,${f_{g}}^{2G}$ is passed to the inter-group Mamba module, and after *M* layer Mamba update, the feature ${f_{g}^{M}}^{2G}$ is obtained, and then the feature pooling is carried out to obtain the global feature *f*. Its structure is shown in Fig. 2(B). The module is mainly composed of two parts:

(1) Intra-group SGTransformer

Conventional Transformer often has a sharp number of parameters with the deepening of the network and the increase of channels. Too many parameters will limit the efficiency of feature extraction and the generalization ability of the model. In order to overcome this limitation, we use Fourier-enhanced grouping Transformer for intra-group feature extraction.

In SGTransformer, in order to further enhance the model's performance on spatial geometric features, we add Fourier transform position transformation to the group. First, the input features are converted into frequency domain through Fourier transform, and then the geometry is enhanced through frequency domain filtering operations. Finally, it is restored to spatial domain through inverse Fourier transform. This augmentation operation can significantly improve the network's ability to capture complex geometries. In addition, the grouping strategy can make the feature extraction more refined, so that each group can focus on learning different sub-features, thereby improving the resolution of feature extraction and the expressive ability of the model. The specific formula is as follows:

First, after performing linear transformation and grouping on the input feature *X*, *q*, *k*_*g*_, *v*_*g*_ are obtained. The specific calculation formulas are as follows:

(1) $$q=W_{Q}X,W_{Q}\in R^{N\times C}$$

(2) $$k_{g}=Group\left(W_{K}X,i,P\right),W_{K}\in R^{N\times C}$$

(3) $$v_{g}=Group\left(W_{V}X,i\right),W_{V}\in R^{N\times C}$$

Here, *W*_*Q*_, *W*_*Q*_, *W*_*V*_ represent linear transformation matrices, respectively, where the input feature $X\in R^{N\times D}$, where *N* represents the number of point features, *D* represents dimensions, *P* represents point coordinates, and *Group*() is a local feature aggregation operation that represents grouping based on the reference index *i*.

Secondly, we make Fourier changes to the position coding, and integrate the high-frequency and low-frequency features into the position coding, so as to enhance the geometric characteristics of the features. Specifically, the Fourier position code is first calculated, and the position coordinates are processed by sine and cosine functions to obtain *p*_sin_, *p*_cos_, respectively,

(4) $$p_{\sin}=sin\left(p_{i},f_{i}\right)$$

(5) $$p_{\cos}=cos\left(p_{i},f_{i}\right)$$

Where *f*_*i*_ ​= ​2^*i*^ means frequency. Then the geometric position encoding *p*_*i*_ is dot multiplied by *v*′ to obtain enhanced spatial geometric features *v*′,

(6) $$v^{\prime }=v⊙pos\left(p_{i},p_{j}\right)$$

(7) $$pos\left(p_{i},p_{j}\right)=MLP\left(p_{i}-p_{j}\right)$$

Finally, the intra-group feature ${f_{g}}^{G}$ is obtained through group weighted fusion, and the specific calculation formula is as follows:

(8) $$w_{ij}=\omega \left(\gamma \left(q_{i},k_{j}\right)\right)$$

(9) $${f_{g}}^{G}=\overset{M\left(p_{i}\right)}{\underset{x_{j}}{\sum }}\overset{g}{\underset{l=1}{\sum }}\overset{c/g}{\underset{m=1}{\sum }}\text{Softmax}\left(W_{i}\right)\cdot v_{j}^{\prime }$$

where *γ* denotes the relational function and $w:R^{c}\to R^{g}$ denotes the grouping weight encoding defined in the next stage.

(2) Inter-group Mamba

For situations where there are a large number of groups, using Transformer to process inter-group dependencies will greatly complicate the model's calculations. In order to solve this problem, we introduced the Mamba module, which can reduce the complexity when dealing with inter-group dependencies. to a linear scale. In addition, since the grouping features are random in arrangement, and random grouping features will cause Mamba to degrade the performance of establishing remote dependencies, therefore, we will first calculate the importance of the grouping features and rearrange them.

In the inter-group mamba, it consists of *M* layer mamba modules. For each grouped ${f_{g}}^{G}$, we add them into ${\left\{f_{g}\right\}}_{g=1}^{G}$, and then through importance sorting, new aggregate features*F*_*m*_ are obtained. The sorted input into the *M* layer mamba is pooled with the maximum features to obtain global features. The specific calculation formula is as follows:

(10) $$\;S_{p}=\text{ReLU}\left(\text{BN}\left(F_{\text{p}}*W_{i}\right)\right)$$

Where, *S*_*p*_ represents the importance score, *F*_p_ represents the feature after the dimension transformation, and *W*_*i*_ represents the weight matrix.

(11) $$z_{m}=\text{DW\_Conv}\circ \text{MLP}\circ \text{LN}\left(F_{m-1}\right)$$

(12) $$F_{n}=\text{MLP}\circ \text{LN}\left(\text{SSM}\left(\sigma \left(z_{m}\right)\right)\right)\cdot \sigma \left(\text{LN}\left(F_{m-1}\right)\right)$$

(13) $$F_{m}=F_{n}+F_{m-1}$$

here,*F*_*m*_ represents the grouping feature of the first *m* layer,*F*_*m*−1_ represents the grouping feature of the previous layer, DW_Conv represents the deep convolution operation, LN represents the LayerNorm operation, SSM represents the state space, in addition, *σ* represents the SiLU activation function and ⋅ represents the dot product operation.

#### 2.4.2 Wavelet grid feature fusion encoder(WGMS)

Downsampling and feature fusion are important means to effectively capture complex morphological features of point clouds. In sampling, the farthest point sampling is usually used (FPS) ​+ ​neighborhood query combination process, which will lead to poor spatial alignment of point sets, and the information density and overlap degree of query point sets cannot be controlled, resulting in insufficient information utilization. In addition, feature fusion usually uses simple feature splicing or weighted averaging, which cannot make full use of different levels of features. In the rubber tree point cloud, the morphological differences between different individuals are large, such as the height of the trunk, the shape of the crown and the distribution of leaves. This irregularity poses a major challenge to point cloud sampling and feature fusion. In order to solve this problem, we propose a wavelet grid feature fusion encoder (WGMS).

The module firstly decomposes the frequency components of the wavelet-variant point cloud features to obtain important low-frequency features (e.g. the height of the trunk and the contour of the crown) and identifies high-frequency detail features (e.g. the distribution of the leaves and the shape of the branches), and then downsamples the augmented point cloud features by using the gridded representation, divides the space into non-overlapping partitions, and further aggregates the neighborhood features. The multilevel feature fusion processes the returned features from different levels of the encoder, and fuses the spatial geometric features at different scales in a hierarchical and progressive manner with a gradual weighting to improve the ability to capture the global spatial information of the model. The structure is shown in Fig. 2(C), the module is divided into two parts, WGrid and MSFF:

(1) Wavelet Grid(WGrid)

First, given the input point cloud feature $F_{w}\in R^{N\times C}$, which is extracted from the intermediate layer of the encoder and refined by a Linear-BN-ReLU operation to encode local contextual information, the input feature is decomposed by wavelet through discrete wavelet changes to obtain different components *c*_*A*_,*c*_*H*_,*c*_*V*_,*c*_*D*_, and then the high-frequency information is enhanced, and the enhanced feature is fused with the original feature to obtain the feature *F*_*i*_ and then the point cloud space is divided into *n*_0_ non-overlapping partitions *M*_*i*_, each partition contains point cloud coordinates and corresponding features, *M*_*i*_ ​= ​*P*_*i*_ ​+ ​*F*_*i*_, *i* ​= ​1, 2 …, *n*_0_, the point coordinate *p*_*i*_ of each partition is averaged pooled, and the point feature *f*_*i*_ is maximally pooled. Finally, the results are aggregated to get the down-sampling *M*′ for the next stage. The specific calculation formula is as follows:

(14) $$\left\{c_{A},\left(c_{H},c_{V},c_{D}\right)\right\}=\text{DWT}2\left(F_{i},w\right)$$

(15) $$F_{i}=F_{w}+\text{IDWT}2\left(\left(c_{A},\left(c_{H}\cdot \alpha ,c_{V}\cdot \alpha ,c_{D}\cdot \alpha \right)\right),\text{w}\right)$$

Where *DWT*2 represents the discrete wavelet transform, *IDWT*2 represents the inverse wavelet transform, *α* represents the enhancement factor, and *w* refers to the type of the discrete wavelet.

(16) $$f_{i}^{\prime }=\text{MaxPool}\left(\left\{Uf_{j}|f_{j}\in F_{i}\right\}\right)$$

(17) $$p_{i}^{\prime }=\text{MeanPool}\left(\left\{p_{j}|p_{j}\in P_{i}\right\}\right)$$

Where $U\in R^{c\times c_{0}}$ refers to the linear projection matrix,$f_{i}^{\prime }\in R^{c_{0}}$ refers to the pooled features, and $p_{i}^{\prime }\in R^{c_{0}}$ refers to the pooled coordinates.

(2) Multi-Scale Feature Fusion(MSFF)

The encoder output of the previous feature fusion is defined as *AM*_*i*−1_, and the point cloud feature of this encoder is defined as *F*_*i*_. First, *F*_*E*_ and *AMSF*_*E*−1_ performs convolution layer, The activation function processing of the batch normalization layer and ReLU produces the fused features *F*_*t*_, and then the weighted average summation is performed to obtain the weight of *ω*, and the weight of *ω* is calculated normalized to [0,1] interval, the weight vector *γ*^*A*^ is obtained, and then the attention weight is applied to the feature *F*_*t*_ to obtain the weighted fusion feature *F*_*A*_.

(18) $$F_{t}=BN\left(Conv1d\left(AM_{i-1},F_{i}\right)\right),\omega =ReLU\left(F_{t}\right)$$

(19) $$\gamma^{A}=\sigma \left(\omega \right),\;\sigma \left(x\right)=\frac{1}{1+e^{-x}}$$

(20) $$F_{A}=F_{t}\times \gamma^{A}$$

Then, we convolve *F*_*A*_to extract spatial information, construct local spatial features through neighborhood *N*(*i*), and use *ReLU*activation function to enhance nonlinear features, obtain spatial features $F_{i}^{S}$, and generate spatial weights $\gamma_{i}^{SA}$to obtain spatially enhanced features *F*_*S*_, and connect *F*_*t*_ with *F*_*S*_ to obtain the final output fusion feature *F*_*out*_.

(21) $$F_{i}^{S}=\text{ReLU}\left({\text{Conv}}_{3\times 3}\left(F_{j}^{A}|j\in N\left(i\right)\right)\right)$$

(22) $$\gamma_{i}^{SA}=\sigma \left({\text{Conv}}_{1\times 1}\left(F_{i}^{S}\right)\right),$$

(23) $$F_{S}=F_{i}^{S}\times \gamma_{i}^{SA}$$

(24) $$F_{\mathrm{out}}=F_{S}+F_{t}$$

Among them, j represents the index of point i in the neighborhood.

The MSFF module accepts the feature output of the previous layer, and after feature fusion, outputs it to the encoding layer. As the network deepens, the hierarchical features of the MSFF module become richer. This improves the model's ability to effectively identify complex features.

#### 2.4.3 Scale optimization algorithm (SCPO)

In deep learning, the learning rate is crucial to the convergence speed and segmentation accuracy of the model, but there is often an imbalance in the learning of different scale features. Larger learning rates tend to learn large-scale features quickly, but tend to ignore small-scale features; smaller learning rates can capture small-scale features in detail, but the overall convergence speed is slower. This imbalance is particularly significant in segmentation tasks, affecting the model's balanced learning of local and global features. To address this problem, we propose the Scale Optimization Coding Algorithm (SCPO). The algorithm combines the properties of particle swarm optimization and hyperbolic sine-cosine function, where the value of cosh is always greater than 1, which helps to locally search for the equilibrium point, and the value of sinh is in [−1, 1] and close to zero, which provides fine-tuning support for the fine search. Through this combination, SCPO achieves dynamic optimization of the learning rate and promotes balanced learning of the model on different scale features.

In SCPO, the iterative process begins by randomizing a set of alternative solutions, which results in a matrix *X* of size *N* ​× ​*dim*. Subsequently, the next position is searched according to the initialized position. In the process of local search optimization, it is mainly divided into two stages: exploration and two stages. First, search the area near the location through the exploration stage, and then gradually approach the optimal solution through the stage. Its structure is shown in Fig. 2(D).

In the first stage, we focus on exploring the potential unknown region, and use the range of hyperbolic sine and cosine as the boundary value to obtain the following position update function:

(25) $$X_{i,j}^{t+1}=\left\{\begin{array}{ll} X_{\mathrm{best}}^{t}-r_{1}\times W_{1}\times X_{\left(i,j\right)}^{t},\; & \text{if ​}r_{2}>0.5\\ X_{\mathrm{best}}^{t}+r_{1}\times W_{1}\times X_{\left(i,j\right)}^{t},\; & \text{if ​}r_{2}<0.5 \end{array}\right)$$

Among them, t represents the current iteration, $X_{i,j}^{t}$ and $X_{i,j}^{t+1}$ represent the position in the current iteration and the next iteration respectively, $X_{\mathrm{best}}^{t}$ represents the position corresponding to the optimal solution in the current stage, *r*1/*r*2 represents a random number located in the cosh function range [0,1], where *W*_1_ represents controlling the current position in the exploration phase, Move away from the initial position to get closer to the optimal solution. W is represented by formula (26):

(26) $$W_{1}=r_{3}\times a_{1}\times \left(\cosh r_{4}+u\times \sinh r_{4}-1\right)$$

(27) $$a_{1}=-3.9\times \frac{t}{\text{Max ​Iteration}}+3\times m$$

Here *a*_1_ is a decreasing function, where *r*3/*r*4 represents the random number in [0,1]. *u* represents a sensitivity coefficient. Used to control the search accuracy of the first stage, equal to 0.388. *m* represents the sensitivity coefficient of the control accuracy of *a*_1_.

**Figure:**
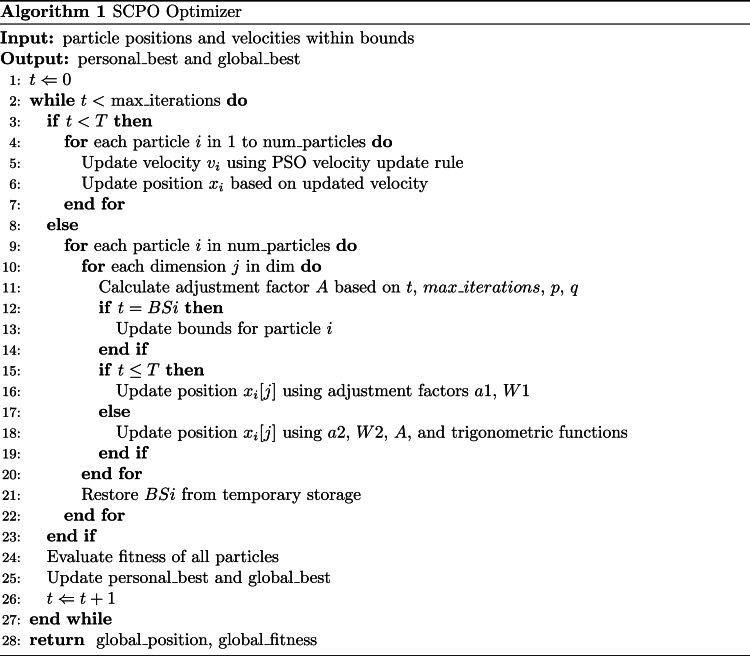

In the second stage, the depth will be carried out around the optimal solution so far, and the intensity of the search will increase with iteration, the update function is shown by equation (28):

(28) $$X_{\left(i,j\right)}^{t+1}=X_{\left(i,j\right)}^{t}+r_{11}\times \frac{\sinh r_{11}}{\cosh r_{12}}\left|W_{2}X_{\mathrm{best}}^{j}-X_{\left(i,j\right)}^{t}\right|$$

(29) $$W_{2}=r_{6}\times a_{2}$$

Among them, *r*_11_/*r*_12_ represents a random number between the interval [0,1], *r*_6_ represents a random number located in [0,1], and *a*_2_ represents a coefficient. *W*_2_ controls the depth in the phase, its absolute value gradually increases with iterations.

During the global search process, the historical optimal position and global optimal position of the particle swarm are used to determine the search direction. The entire space is explored through the position and speed of a single particle. In addition, under the influence of cognitive learning factors and social learning factors, the search range of the particle swarm will be expanded to ensure that the entire particle population can find the global optimal solution, (23), (23) and equation (24), (24) describe the update part of speed and position respectively.

(23) $$V_{i}\left(t+1\right)=c_{1}r_{1}\left(p_{i}\left(t\right)-Z_{i}\left(t\right)\right)+c_{2}r_{2}\left(G\left(t\right)-Z_{i}\left(t\right)\right)$$

(24) $$Z_{i}\left(t\right)=Z_{i}\left(t\right)+V_{i}\left(t\right)$$

Where *w*denotes inertia weights, *c*_1_denotes individual learning factors, *c*_2_denotes social learning factors, *r*_1_and *r*_2_are random numbers in the range [0, 1], where *p*_*i*_denotes the most familiar position of a single particle.

The SCPO algorithm combines the global search ability of particle swarm with the local optimization ability of SCHO, and effectively extracts the optimal solution through the ability of SCHO to balance the search boundary. By this form, the performance and robustness of the model is significantly improved. For better understanding of the algorithm, we provide pseudo-code about SCPO, as shown in the Algorithm 1.

## 3 Experiment and analysis

This section is mainly divided into four parts: (1) Data set preparation; (2) Setting of experimental environment and hyperparameters; (3) Definition of evaluation indicators; (4) Effectiveness of each module in TM-WSNet, and comparative analysis with other models.

### 3.1 Dataset processing

Self-built dataset:To verify our effectiveness, we use the rubber tree data set we collected and established ourselves.

(1) RubberTree: We independently collected and organized the rubber tree data set from the Rubber Research Institute of the Academy of Tropical Agricultural Sciences in Danzhou, Hainan Province. It consists of 990 independent rubber trees. Each data file contains two categories: rubber tree tree points and background points.

Public datasets:In order to verify our effectiveness, we used the widely used ShapeNetPart Dataset [38], the graph-based leaf–wood separation (GBS) dataset [39] and ForestSemantic [40] in the experiment.

(1) ShapeNetPart: is a data set specifically used for 3D point cloud segmentation tasks. It contains 16 types of objects, such as airplanes, chairs, tables, etc. Each type of object is divided into multiple semantic parts. There are a total of 50 segmentation parts. Each object is composed of It consists of about 2048 points, each point contains spatial coordinates x, y, z and semantic segmentation labels.
(2) GBS: It is a single tree data set covering tropical, temperate, and boreal regions [39]. We selected 56 single trees of different types to segment between trunks and crowns.
(3) ForestSemantic: This dataset provides detailed annotations of typical objects in forest scenes, including ground, shrubs, trunks, branches, and leaves. To meet the basic experimental requirements, we adopted the preprocessing method from our self-built dataset RubberTree and applied the same processing steps to the ForestSemantic dataset. First, the treeiso method was used to perform initial coarse segmentation, where background noise points and ground points were grouped into the same category. Given the varying difficulty of extracting individual targets from different land parcels in the ForestSemantic dataset, we selected 84 initial data samples that were able to successfully isolate a single target individual and further constructed refined training samples. This ensured that the proposed method could accurately capture and differentiate the target individual. Finally, we employed common data augmentation techniques such as rotation, scaling, and flipping to create a dataset containing 252 independent samples.

### 3.2 Experimental environment and prameter settings

#### 3.2.1 Experimental environment

The experimental environment was defined as follows: the hardware used consisted of an Intel(R) Xeon(R) Platinum 8362 CPU with an NVIDIA RTX 3090GPU; while the versions of Python and CUDA did not affect the results of the experiments, they had to be compatible with the specific software and hardware used. Therefore, TM-WSNet was implemented using PyTorch 1.11.0 and Python 3.8 with the following versions: Ubuntu 20.04 for the OS and 11.8 for the CUDA Toolkit.

#### 3.2.2 Prameter settings

In this study, the choice of hyperparameters is crucial for the results. To select the experimental hyperparameters, we set the search range for the initial learning rate to 1e-5 to 1e-8 and the batch to 4. During the experiment, the training period and the size of the grid are determined through the experiment.

**Fig. 3:**
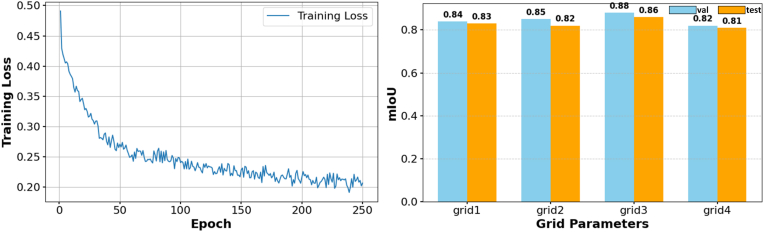
(a):loss changes when the number of training rounds is 200,(b):Four different grids size, effect on mIoU.

In order to obtain the training period, we trained 250 epochs, as shown in Fig. 3. When the model is in 200 rounds, the loss of the entire network is basically close to convergence stability, so we decided to set the training period of the network model to 200.

In the model initialization feature grouping stage, the grid size setting of point cloud parameters determines the grouping of point cloud data at each processing stage and how to effectively aggregate and encode points in local areas. A reasonable mesh size can help the model extract effective local geometric features while perceiving the global structure. Here we tested four different grids, respectively set to grid_1: (0.005, 0.0125, 0.025, 0.0625), grid_2: (0.01, 0.025, 0.05, 0.125), grid_3: (0.01, 0.025, 0.1, 0.25), grid_4:(0.02, 0.05, 0.1, 0.25). By comparing the experimental result Fig. 3, we found that when the grid size is too small (such as grid_1), although the detail capturing ability is relatively strong, but its performance is insufficient in extracting global structural information, which can easily lead to the model overfitting of local details. When the grid size is too large (such as grid_4), the model tends to ignore the details of local geometric features, resulting in a decrease in segmentation accuracy. Grid_3 reflects the feature extraction process from fine-grained to coarse-grained in the experiment. This setting can effectively extract features at different granularity levels of the rubber tree point cloud, which not only ensures the precise extraction of fine-grained features, but also enhances the model's perception of the global structure, thus ensuring the segmentation performance of the model.

### 3.3 Experimental indicators

In this paper, we use the mean intersection and merger ratio (mIoU), mean accuracy (mAcc), mean F1 score (mF1), mean precision (mPrec), mean recall (mRec), and the *R*^2^ metrics to evaluate the performance of TM-WSNet in the rubber tree single tree segmentation task. In the semantic segmentation task, mIoU is implemented to evaluate the overall performance. mAcc denotes the overall correct prediction rate of the model on all categories, i.e., the ratio of the number of correctly predicted pixels to the total number of pixels. mF1 provides an overall assessment of the model's performance by considering the average precision and the average recall. mPrec measures the model's precision. mRec evaluates the model's overall performance, and in addition. We adopt *R*^2^ to evaluate the fitting effect of the model, and assess the consistency of the spatial distribution of the model in monoki segmentation by calculating the correlation between the predicted values and the true values. The specific formula is as follows:

(30) $$mIoU=\frac{1}{N}\overset{N}{\underset{i=1}{\sum }}\frac{TP_{i}}{TP_{i}+FP_{i}+FN_{i}}$$

(31) $$mAcc=\overset{N}{\underset{i=1}{\sum }}\frac{TP_{i}}{\left(TP_{i}+FP_{i}+FN_{i}+TN_{i}\right)}$$

(32) $$mF1=\overset{N}{\underset{i=1}{\sum }}\frac{2\cdot Prec_{i}\cdot Rec_{i}}{Prec_{i}+Rec_{i}}$$

(33) $$mPrec=\frac{1}{N}\overset{N}{\underset{i=1}{\sum }}\frac{TP_{i}}{TP_{i}+FP_{i}}$$

(34) $$mRec=\frac{1}{N}\overset{N}{\underset{i=1}{\sum }}\frac{TP_{i}}{TP_{i}+FN_{i}}$$

(35) $$R^{2}=1-\frac{\sum_{i=1}^{n}{\left(y_{i}-{\hat{y}}_{i}\right)}^{2}}{\sum_{i=1}^{n}{\left(y_{i}-\bar{y}\right)}^{2}}$$

Where *N* denotes the total number of categories. *TP*_*i*_, *FP*_*i*_,*FN*_*i*_ denote the true, false positive and false negative examples of the ith category, respectively. *y*_*i*_ denotes the true value, ${\hat{y}}_{i}$ denotes the model predicted value, $\bar{y}$ denotes the mean of the true value, and *n* denotes the sample size.

### 3.4 Experimental results and analysis

#### 3.4.1 Performance experiments

**Fig. 4:**
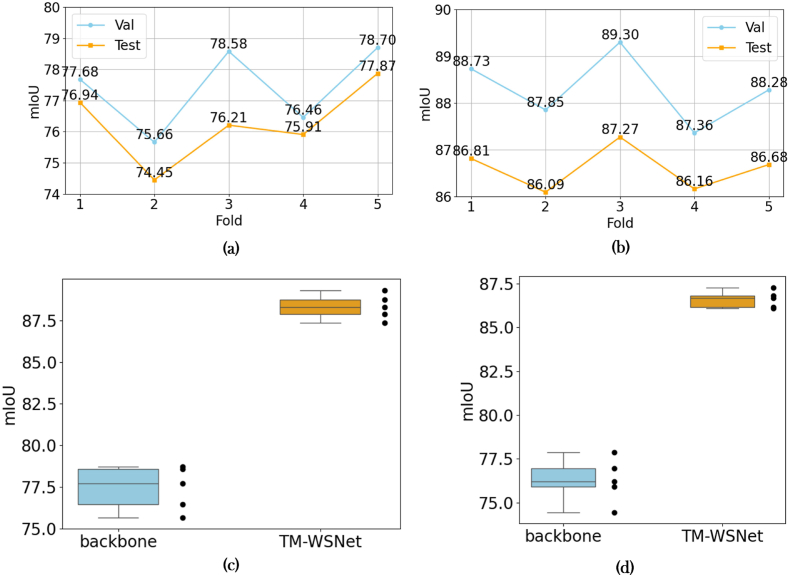
(a): Results of backbone model,(b):Results of TM-WSNet model,(c):The comparison results on the validation dataset,(d):The comparison results on the test dataset.

In this experiment, we randomly arrange the rubber tree point cloud dataset, and divide the dataset into training set and test set with a size of 4:1. Compared with the TM-WSNet model, the only difference between the backbone network is the removal of SGTramba, WGMS and SCPO. In order to evaluate the performance and stability of the model, the backbone and TM-WSNet models were trained separately using 5-fold cross-validation. The experimental results show that Fig. 4, TM-WSNet has more accurate segmentation accuracy, and the average mIoU of the five sets of experimental results generated reaches 90.35 ​%, and the data fluctuation of mAcc is small, which shows that TM-WSNet has stronger segmentation ability and higher stability compared with the benchmark model.

#### 3.4.2 Method effectiveness experiments

In order to verify the effectiveness and advancement of the three proposed models, the following experiments are carried out on the SGTramba, WGMS, and SCPO modules of the TM-WSNet model on a self-built rubber tree dataset. Under the same experimental facilities, the effectiveness of each innovative module is verified.

(a) Effectiveness of SGTramba

The SGTramba module proposed in this paper uses a hybrid feature extraction mechanism to accurately extract the key part of the point cloud features, thus improving the accuracy of the model, and uses a grouped transformer to reduce the energy consumption of the model in the calculation process. The serious overlap and background interference problems in rubber tree segmentation tasks are effectively dealt with. To fully evaluate the performance of the SGTramba module, we compared it with CAM [41], SA [42], Transformer [11], GTransformer [43], PointMamba [20] and other modules were compared. Specifically, the above modules were incorporated into TM-WSNet network to replace SGTramba for training, and the performance of different modules under the same network architecture was compared.

From the experimental Table 1, we can see that the SGTramba module reached 83.12 ​% on the mIoU, compared with other modules such as Transformer (82.11 ​%) and GTransformer(82.62 ​%) has a significant improvement. Compared with the SA module, the SGTramba module shows obvious advantages in both intra-group detail capture and inter-group dependence modeling.

**Table 1:** Comparison of SGTramba, WGMS, SCPO and other feature extraction modules.

type	Method	mIoU(%)	mAcc(%)	mPrec(%)	mF1(%)	mRecall(%)
SGTramba	SA	79.98	84.64	88.88	93.56	84.64
	CAM	80.20	91.23	89.01	86.90	90.52
	Transformer	82.11	90.51	90.27	90.17	89.99
	GTransformer	82.62	90.42	90.65	90.46	90.51
	PointMamba	82.96	90.74	90.83	90.68	90.62
	SGTramba	**83.12**	90.69	**90.95**	**90.76**	**90.84**
WGMS	WGrid ​+ ​backbone	81.45	90.38	89.84	89.77	89.68
	MSFF ​+ ​backbone	83.26	90.70	91.05	90.85	91.03
	WGrid ​+ ​MLP_blocks	82.33	90.41	90.44	90.29	90.20
	WGrid ​+ ​MSG	83.30	90.94	91.03	90.88	90.81
	WGMS	**84.48**	**91.76**	**91.70**	**91.58**	**91.45**
SCPO	Adam	79.19	89.15	88.43	88.38	88.43
	PSO	80.38	88.96	89.34	89.10	89.27
	SCHO	80.99	90.24	89.55	89.49	89.50
	SCPO	**81.82**	**90.54**	**90.07**	**89.99**	**89.87**

The comparison results reveal that the SA module, which focuses on local feature extraction, demonstrates limited performance due to its weak capability in capturing global contextual information. Similarly, the CAM module, which applies attention solely along the feature channels, fails to fully exploit the spatial geometric structure of the point cloud, resulting in suboptimal segmentation performance in complex scenes. In contrast, both the Transformer and GTransformer modules enhance global feature modeling through attention mechanisms, leading to significant improvements across all evaluation metrics and demonstrating stronger feature representation capabilities. PointMamba further integrates spatial structure awareness with sequence modeling advantages, thereby improving contextual understanding and achieving better segmentation performance than the Transformer. Notably, the SGTramba module achieves the best overall performance, confirming its effectiveness and superiority in integrating both local geometric features and global contextual information.

(b) Effectiveness of WGMS

The WGMS module extracts the point cloud features of different levels through the fusion operation of wavelet grid and multi-level features, and performs dynamic weighted fusion of different shape features, so as to effectively solve the problem of irregular tree shape in the process of rubber tree segmentation. To verify the effectiveness of the WGMS module, we compared it with the MSG [28] module and MLP_blocks [44], incorporating different modules into the model for training and evaluation.

Experimental results as shown in Table 1 demonstrate that the WGMS module outperforms all other modules across various evaluation metrics, achieving an mIoU of 84.48 ​% and an mAcc of 91.76 ​%. The superior performance of WGMS can be attributed to its adaptive feature fusion mechanism and wavelet grid pooling-based feature enhancement, which together enable more effective processing of point cloud features.

The experimental results indicate that the WGrid ​+ ​MSG module is limited to extracting features from a single hierarchical level and lacks the ability to dynamically adjust the weights across multiple feature scales. This limitation results in slightly lower segmentation accuracy compared to WGMS. The MLP_blocks module, while possessing strong nonlinear mapping capabilities, disregards the spatial positioning of points in the point cloud. As a result, it is prone to information loss when handling complex 3D structures, leading to subpar segmentation performance. In contrast, the WGMS module effectively integrates geometric features across multiple levels, enhancing the spatial geometric representation of features. This comprehensive feature representation mitigates the risk of missing important spatial relationships by relying solely on point-level features. Consequently, WGMS demonstrates superior robustness and segmentation performance in 3D point cloud tasks.

(c) Effectiveness of SCPO

Aiming at the problem of different learning rates at different scales, we propose a scale optimization algorithm to optimize the learning rates of the model at different scales. In order to verify the effectiveness of SCPO, we compared it with PSO [45], SCHO [46] and Adam optimizer [47]. The experimental results are shown in Table 1.

Experimental results show that SCPO achieves a mean Intersection over Union (mIoU) of 81.82 ​%, demonstrating superior segmentation accuracy compared to SCHO and PSO. PSO, as a population-based optimization algorithm, tends to explore a wide search space, which often results in insufficient local exploitation. On the other hand, SCHO is prone to premature convergence during the search process, frequently getting trapped in local optima and returning suboptimal results, thereby compromising the overall learning capability and final accuracy of the model. In contrast, SCPO effectively integrates the respective strengths of both PSO and SCHO, achieving a better balance between exploration and exploitation. This leads to more efficient learning rate optimization and improved model performance.

#### 3.4.3 Ablation experiment

We test the proposed module on the RubberTree dataset and verify its effectiveness. We trained the backbone network with the same hyperparameters in the RubberTree dataset for 200 epochs, and selected the best-performing model in the validation set. During the experiment, we used control variables to gradually integrate SGTRamba, WGMS and SCPO, and designed eight sets of ablation experiments.

The experimental results are shown in Table 2, which indicate that SGTramba, WGMS and SCPO in TM-WSNet have a significant effect on the enhancement of the segmentation ability of the model. By comparing Backbone with Backbone ​+ ​SGTramba group, we find that adding SGTramba module improves the mIoU by 3.93 ​%, with mAcc improving the most significantly, which reflects the excellent performance of SGTramba in carrying out feature extraction. Comparing Backbone with WGMS group, we find that adding WGMS yields an improvement of 5.29 ​% in mIoU and 3.20 ​% in mF1, indicating that WGMS is effective in performing multilevel feature aggregation and improves the overall segmentation accuracy of the model. In addition, comparing the Backbone and SCPO groups, we know that the values of each evaluation parameter of the model are effectively improved when optimised using the SCPO algorithm, indicating that SCPO enables the learning rate of the model to converge effectively, allowing the model to reach a higher level of performance. Finally, in comparing TM-WSNet with the Backbone model, we are able to obtain that TM-WSNet outperforms the Backbone model in all parameter accuracies, with a 9.59 ​% increase in mIoU, a plus 5.35 ​% increase in mAcc, and a 6.12 ​% increase in mPrec, and these results indicate that TM-WSNet has a significant performance in the rubber tree point cloud segmentation task with significant performance advantages.

**Table 2:** Ablation experiment of TM-WSNet.

Method	mIoU(%)	mAcc(%)	mPrec(%)	mF1(%)	mRecall(%)
Backbone	79.19	89.15	88.43	88.38	88.43
+SCPO	81.82	90.54	90.07	89.99	89.87
+SGTramba	83.12	90.69	90.95	90.76	90.84
+WGMS	84.48	91.76	91.70	91.58	91.45
SGTramba ​+ ​SCPO	86.55	92.83	92.79	92.75	92.30
WGMS ​+ ​SCPO	87.81	94.06	94.01	94.22	93.22
WGMS ​+ ​SGTramba	88.28	94.29	93.54	95.12	93.50
TM-WSNet	**88.78**	**94.50**	**94.55**	94.59	**93.52**

#### 3.4.4 Visualize results comparison

**Fig. 5:**
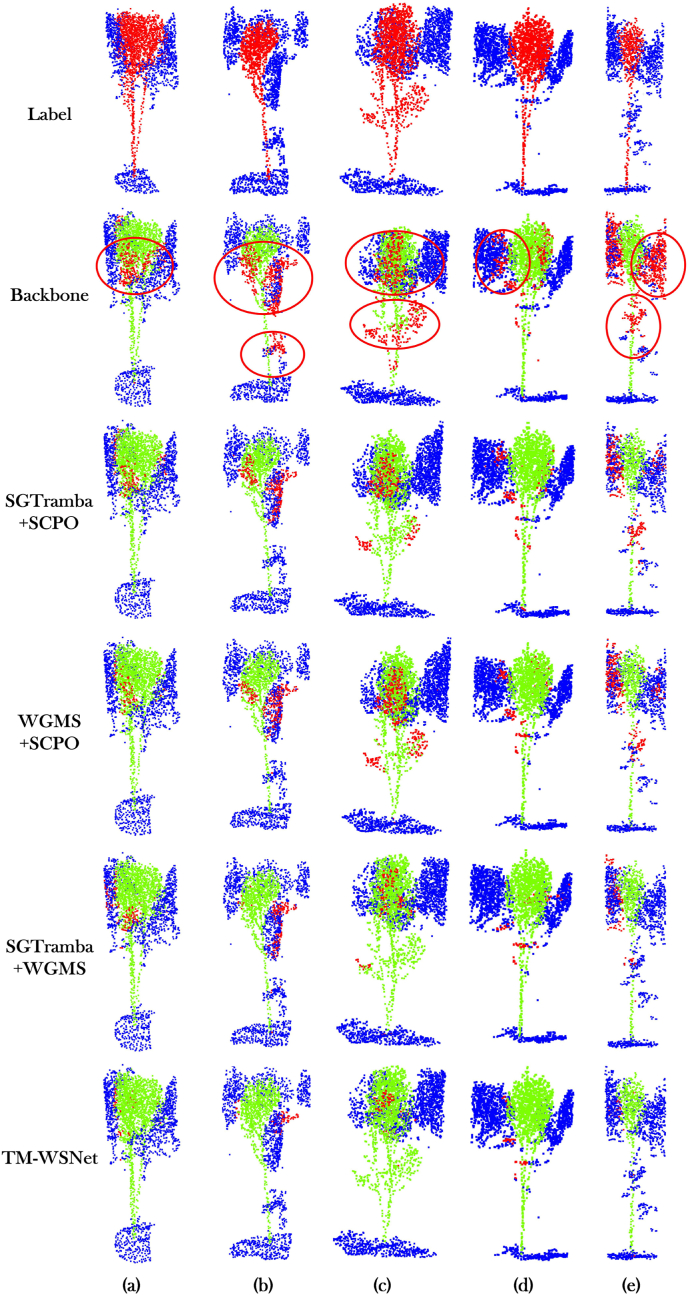
The comparison of the effectiveness of feature extraction of each module in the TM-WSNet model, where the green part indicates the correctly predicted tree point, the blue part is the correctly predicted other points, and the red part is the wrong point. Red circles mark regions with frequent segmentation errors.

In order to be able to visually evaluate the degree of contribution of each module to the TM-WSNet model for rubber tree point cloud segmentation. We select five representative point cloud samples from the test set of the ablation experiment for visualization and comparison. The visualization results are shown in Fig. 5, in which we can see that the rubber tree point cloud is divided into three parts, the green domain represents the rubber tree point cloud, the blue domain represents the ground and other unknown points, and the red domain is the prediction error points. During the experiment, we used the control variable method to modularize the combination for SGTramba, WGMS and SCPO and generated 5 different comparison groups. By observing Fig. 5(c,d), it is found that SGTramba achieves accurate segmentation of rubber tree trunks by significantly enhancing the model's ability to differentiate between the ground and other points compared to Backbone. This indicates that by using the SGTramba module, the TM-WSNet model is more refined in the processing of point cloud features, especially in the representation of spatial geometric features is effectively improved. And the accuracy of the model is further improved when working together with WGMS, for example, Fig. 5 SGTramba ​+ ​WGMS corresponding to (b). Overall, the SGTramba module is able to effectively capture both local and global features, while the WGMS module further strengthens the global semantic understanding of the model through multi-level feature aggregation, resulting in more accurate segmentation of complex point cloud scenes. Combining the effects of both, the model's performance in rubber tree point cloud segmentation is significantly improved. Therefore, from the experimental results, the TM-WSNet model proposed in this paper shows good performance in the rubber tree segmentation task, especially in the feature recognition of canopy morphology and tree height difference cited in the case of canopy background confusion.

#### 3.4.5 Compare with SOTA methods on RubberTree dataset

In order to verify the sophistication of the TM-WSNet model, we compared the model with state-of-the-art point cloud segmentation networks, including GDANET [48], SPOTR [49], PointNext [50], PointTransformerV2 [43], PointMamba [19], PC-Mamba [20] point cloud model. We perform 200 rounds of training on all models and select the models with performance from the test set of each. To ensure fairness and considering the significant impact of hyperparameters on model performance, we used public code with default hyperparameters to implement the above networks.

We conducted comparative experiments on the RubberTree dataset, with detailed results presented in Table 3. The proposed algorithm outperforms all other methods across evaluation metrics, achieving an mIoU of 88.78 ​%, an mAcc of 94.50 ​%, and an in metric with an *R*^2^ value of 0.9847.

**Table 3:** Comparison results on the RubberTree, ForestSemantic, GBS and ShapeNetPart dataset.

Dataset	Method	mIoU(%)	mAcc(%)	mPrec(%)	mF1(%)	mRecall(%)	*R*^2^
RubberTree	GDANET(2020)	69.91	73.89	79.29	70.43	74.12	0.9388
	SPOTR(2023)	71.76	83.21	83.65	80.58	83.91	0.9485
	PointNext(2022)	72.28	85.91	83.68	82.00	84.51	0.9464
	PTv2(2022)	80.21	90.27	89.06	88.72	90.13	0.9671
	PointMamba(2024)	86.57	91.36	92.89	93.42	94.27	0.9750
	PC-Mamba(2024)	87.15	92.20	93.14	94.09	93.31	0.9788
	TM-WSNet	**88.78**	**94.50**	**94.55**	**94.59**	93.52	0.9847
ForestSemantic	GDANET(2020)	57.35	71.59	72.90	74.26	71.59	0.7522
	SPOTR(2023)	59.82	73.97	76.72	74.38	80.10	0.7536
	PointNext(2022)	61.79	75.84	77.16	76.20	77.23	0.7617
	PTv2(2022)	62.07	76.38	77.04	76.49	76.64	0.7703
	PointMamba(2024)	63.12	76.50	78.73	77.09	80.90	0.7916
	PC-Mamba(2024)	66.59	82.95	80.02	77.28	82.95	0.8294
	TM-WSNet	**66.78**	81.17	**80.08**	**79.02**	81.17	0.8335
ShapeNetPart	GDANET(2020)	75.31	78.64	83.12	78.87	78.44	
	SPOTR(2023)	78.25	83.13	87.98	91.19	83.47	
	PointNext(2022)	82.45	89.78	90.12	89.67	90.21	
	PTv2(2022)	85.43	91.98	94.10	91.98	91.96	
	PointMamba(2024)	85.63	91.37	94.22	92.10	92.90	
	PC-Mamba(2024)	86.67	87,86	93.03	95.59	88.06	
	TM-WSNet	**86.86**	87.78	92.97	**96.81**	87.78	
GBS	GDANET(2020)	69.28	72.12	80.25	70.67	72.33	
	SPOTR(2023)	70.61	78.89	82.77	87.07	78.89	
	PointNext(2022)	70.57	80.35	80.64	75.64	81.35	
	PTv2(2022)	76.19	82.18	86.49	91.27	82.18	
	PointMamba(2024)	77.47	88.77	87.30	85.89	88.77	
	PC-Mamba(2024)	78.95	90.12	87.73	84.59	91.12	
	TM-WSNet	**79.23**	89.35	**88.41**	**87.49**	89.35	

**Fig. 6:**
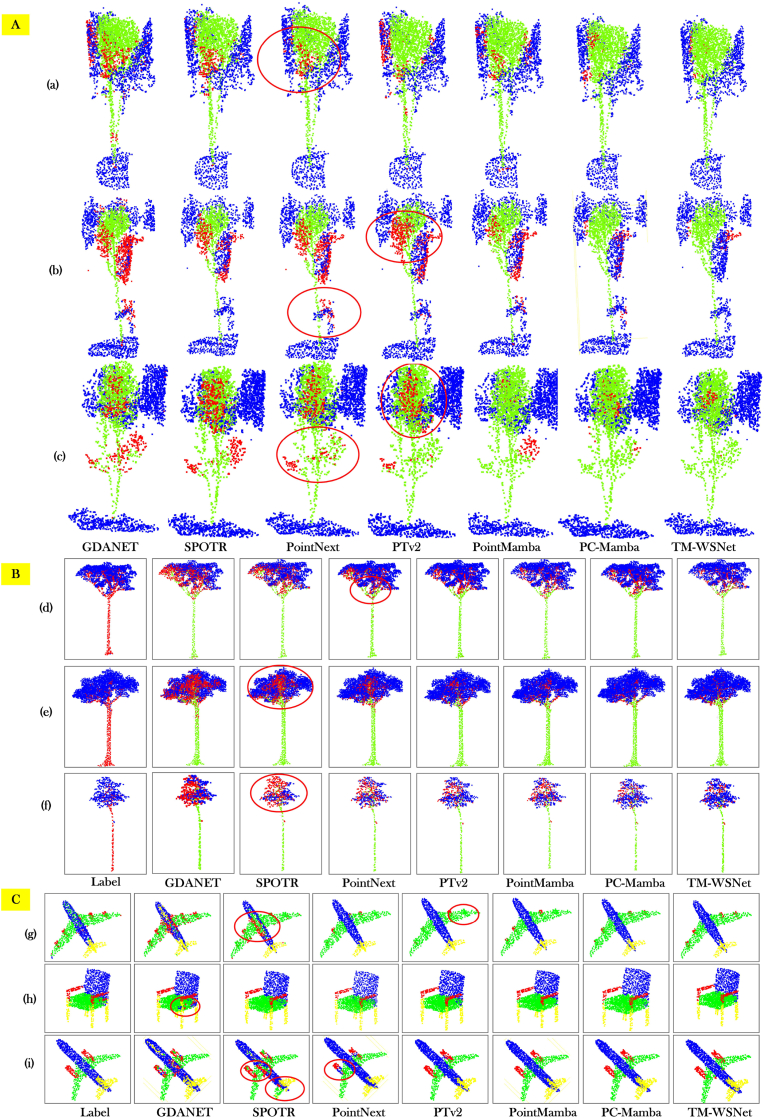
Segmentation results on RubberTree (A), GBS (B), and ShapeNetPart (C) datasets. In (A), green denotes tree points, blue non-tree points, and red misclassified points. In (B) models, blue shows leaf points, green trunk points, and red misclassified areas. Different colors in (C) represent different categories. Red circles mark regions with frequent segmentation errors.

In the visualization results shown in Fig. 6, red points represent misclassified areas, green points indicate correctly segmented rubber tree points, and blue points correspond to other correctly identified points. As observed in Fig. 6(A)(b), the PTv2 model produces a large number of misclassifications, particularly in the background regions of the rubber tree canopy, indicating insufficient feature extraction. In contrast, the visual comparisons between PointMamba and TM-WSNet reveal that our model achieves significantly better performance in handling the boundary regions of the rubber tree canopy. Moreover, in non-canopy areas—such as the sub-canopy region shown in Fig. 6(A)(c) our model demonstrates a clear advantage.

#### 3.4.6 Compare with SOTA methods on the ForestSemantic dataset

To further validate the effectiveness of our method in the field of tree segmentation, we conducted experiments on the ForestSemantic dataset and performed a comparative analysis with the current state-of-the-art (SOTA) methods. The hyperparameters were set as follows: batch size ​= ​2, the search range for the optimizer was set between 1e-8 and 4.9e-5, We trained all models for 200 epochs to ensure the reliability of the experimental results.

From the experimental results in Tables 3 and it can be seen that, on the ForestSemantic dataset, our model outperforms the current SOTA methods in terms of mIoU. Compared to the PTv2 model, our method improves mAcc by 4.79 ​%; in terms of the sample structure parameter R^2^, our model achieves 0.8335, which is significantly better than existing methods. Additionally, although our model performs slightly worse than the PC-Mamba model in terms of mAcc, with a 0.58 ​% lower value, it still shows a clear advantage in overall evaluation metrics and structural parameters, specifically improving mIoU by 0.19 ​% and R^2^ by 0.0041.

**Fig. 7:**
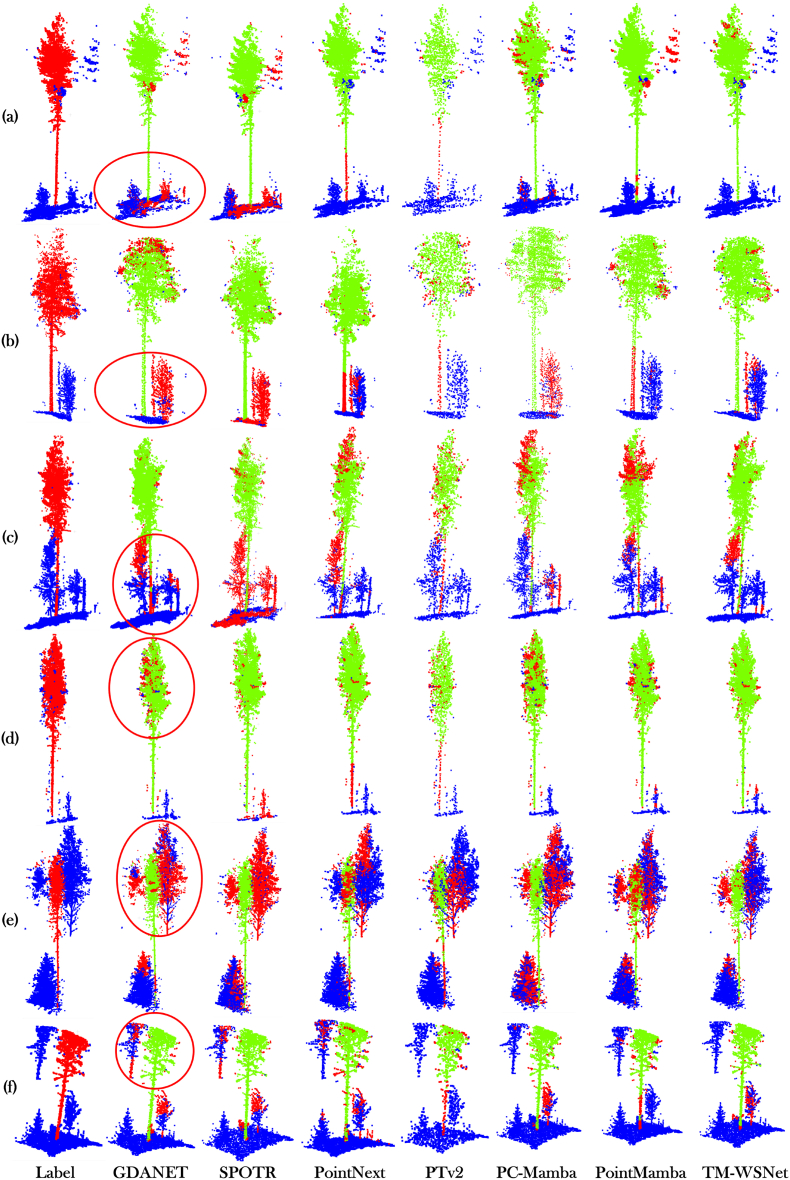
The segmentation results for the ForestSemantic dataset are shown. In the Label column, red represents target point clouds, and blue represents background noise. In other models, green indicates correctly segmented tree point clouds, blue indicates background noise, and red indicates misclassified point clouds. Red circles highlight areas with likely misclassified red points.

Fig. 7 presents some of the visualization results on the ForestSemantic dataset. As shown in the figure, the SPOTR model struggles to effectively separate the ground point cloud in complex near-ground environments. Specifically, from Fig. 7(a), (b), and (c), it is evident that there are numerous misclassified points in the near-ground area, particularly in the transition region between the ground points and the target trees. Further observations in Fig. 7(a), (b), and (d) reveal that both the PointNext and PTv2 models exhibit misclassification during the trunk extraction process. The confusion between trunk points and near-ground background noise is particularly prominent. This suggests that both models may have failed to effectively distinguish between the trunk and near-ground background noise during the feature extraction process, which impacts the segmentation accuracy. Additionally, by examining Fig. 7(c), (d), and (e), we found that the PointMamba model tends to introduce canopy background noise when extracting tree crown features, causing some crown areas to be misclassified as background noise. This issue may stem from the model's incomplete differentiation between the crown and background regions during the feature extraction process. In contrast to the above methods, the visualization results of the TM-WSNet model show that, although misclassification still occurs in some areas, the misclassified points are primarily concentrated in regions where the canopy background noise and target tree points are too similar. Therefore, the TM-WSNet model outperforms the other models in terms of overall segmentation performance, achieving more accurate separation between trees and background regions, especially in complex environments. This indicates that the model exhibits strong robustness.

#### 3.4.7 Compare with SOTA methods on the ShapeNetPart and GBS dataset

Table 3 shows the comparison results of the two datasets, and it can be seen that our model performs well on both the ShapeNetPart dataset and the GBS dataset, and outperforms the others in terms of metrics such as mIoU and mF1. On the ShapeNetPart dataset mIoU reaches 86.86 ​% and mF1 reaches 96.81 ​%; on the GBS dataset, the mIoU of our model reaches 79.23 ​%, and the mF1 reaches 87.49 ​%, and the experimental results show that the TM-WSNet has universality with respect to the point cloud segmentation domain.

The visualization results of GBS dataset and ShapeNetPart dataset are shown in Fig. 6B, 6C. Among other models, GDANET performs feature extraction by employing graph neural network (GNN). It focuses on extracting the global features of the point cloud data, but performs poorly in the local detail part. For example, Fig. 6C(g,i), a large number of wrongly detected points appear at the junction of the airplane wing and fuselage. PointNext implements an improvement of PointNet++, which enhances the feature representation capability by introducing more complex aggregation and feature fusion modules. However, its performance in feature extraction is weak. By comparing PTv2 with the TM-WSNet model, we find that PTv2 is able to effectively handle global contextual features and improve the robustness of the model in dealing with complex scenes. However, observing Fig. 6(i) and comparing it with TM-WSNet, we find that PTv2 is inadequate in modeling local point cloud features and faces significant challenges in segmentation accuracy. And observing the corresponding graphs of PointMamba and PC-Mamba Fig. 6B(d,e,f) we find that Mamba successfully captures the global dependencies of the point cloud through the powerful state-space model and obtains better segmentation results.

Upon examining the visualization results of the TM-WSNet model, we find that it not only achieves outstanding performance in rubber tree segmentation under complex background interference, but also demonstrates competitive results on large-scale, multi-class datasets when compared with state-of-the-art models. Furthermore, TM-WSNet also performs well on small-scale datasets involving complex boundaries between tree canopies and trunks, highlighting its robustness and generalization capability across diverse segmentation scenarios.

### 3.5 Discussion and shortcomings

In order to validate the segmentation of the model in a real environment, we collected brand new point cloud rubber tree data to compare the performance metrics of TM-WSNet with the skeleton model. The data were obtained by scanning the rubber plantation in Danzhou City, Hainan Province, using the LiDAR system of DJIM300RTK UAV.

Experimental results demonstrate that TM-WSNet exhibits clear advantages in real-world environments, particularly in segmenting rubber trees with varying heights and complex structures. Compared to the baseline backbone network, TM-WSNet achieves an mIoU of 86.15 ​%, an mF1 score of 92.55 ​%, and an mAcc of 92.56 ​%.

**Fig. 8:**
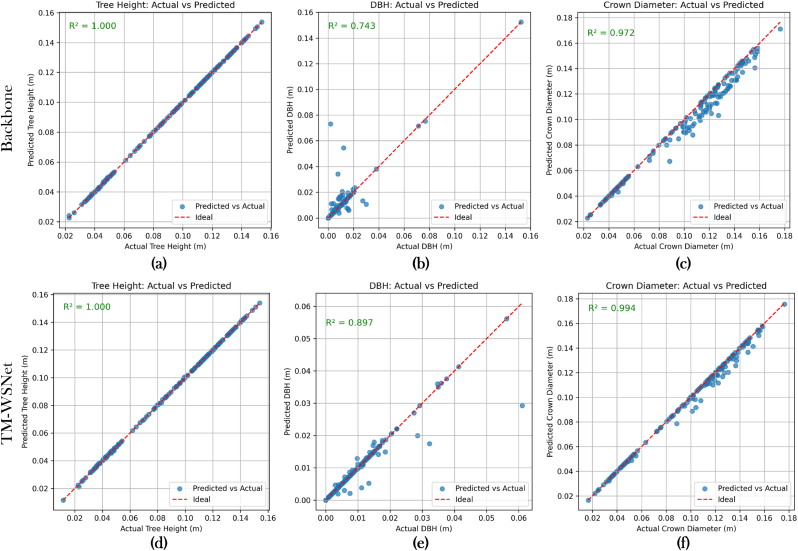
In practical application, the residual distribution map of tree height, DBH and crown diameter(A). In A, (a,b,c) are Backbone comparison results and (d,e,f) are TM-WSNet comparison results.

In addition, we analyse the structural parameters of the rubber tree for the error between the true value and the predicted value, and analyse the results as shown in Fig. 8A. We used the comparative analyses using the three metrics of Tree Height, diameter at breast height (DBH), and Crown Diameter, respectively. Observing the charts Fig. 8A, it can be seen that the *R*^2^ of the TM-WSNet parameter DBH increased by 0.154 compared to the backbone, Crown Diameter's *R*^2^ increases by 0.022, indicating that our model can effectively segment rubber trees and provide a new feasible method for the extraction of structural parameters of rubber trees.

**Fig. 9:**
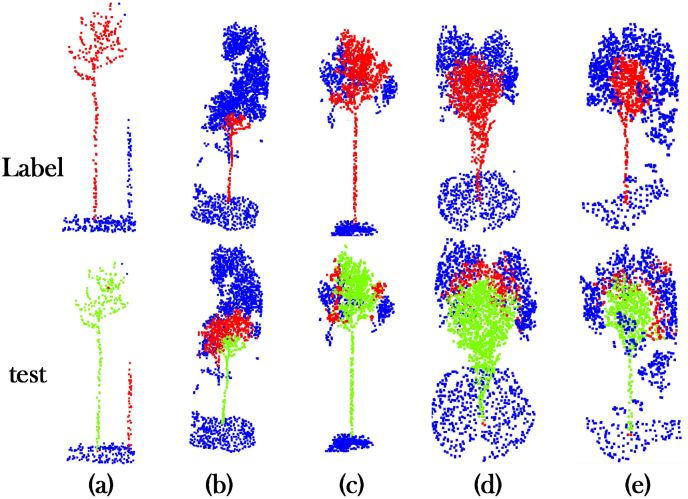
Samples with obvious errors in test results.

However, there is still room for further optimization in the rubber tree segmentation task. As shown in the sample in Fig. 9(a), the model misidentifies background noise with regular shapes as part of the rubber tree trunk, which directly reduces the accuracy of sample parameter estimation. In addition, when rubber tree samples have relatively low heights and their canopy regions overlap or are confused with those of surrounding vegetation—as in the sample shown in Fig. 9(b), the segmentation performance of the model suffers significant deviation. For samples with blurred canopy boundaries, as illustrated in Fig. 9(c) and (d), the model still exhibits a certain degree of misclassification when handling these fuzzy boundaries. Although the above issues limit the model's generalization ability and application accuracy in practical forestry scenarios to some extent, in most standardized rubber plantation operations, the model can still efficiently perform large-scale preliminary segmentation and data collection of rubber trees, thanks to its high automation and relatively stable baseline recognition performance. This demonstrates the model's significant practical value in improving the efficiency of rubber tree resource monitoring and supporting digital forest management.

## 4 Conclusion

To address the challenge of fine-grained segmentation of individual rubber trees in complex environments, this paper proposes a novel point cloud segmentation network named TM-WSNet.

1. In the feature extraction stage, we introduce a hybrid feature extraction module called SGTramba. This module employs grouped Transformers to capture local features and integrates Fourier transform to process spatial features. Meanwhile, Mamba is used to capture global dependencies between different sequence groups, effectively enhancing the model's ability to recognize subtle differences in spatial geometric positions of feature points. For feature enhancement, we design the WGMS module, which uses wavelet-enhanced grid pooling to sample the initial point cloud and adaptively fuses multi-level point cloud features, enabling the model to efficiently handle global contextual information. Finally, we propose a scale-aware optimization algorithm (SCPO) to help the model accurately obtain optimal learning rates across multiple scales, further improving segmentation accuracy.
2. Experimental results demonstrate that TM-WSNet outperforms existing mainstream segmentation networks in the rubber tree segmentation task across all optimization metrics. Compared with PTv2, the proposed model shows better performance in global feature extraction (with mIoU increased by 8.57 ​%, mAcc by 4.53 ​%, and mF1 by 6.87 ​%). Compared with PointMamba, the model better captures boundary features and improves segmentation accuracy (with mIoU increased by 2.21 ​%, mAcc by 3.14 ​%, and mF1 by 1.17 ​%). When compared to PC-Mamba, the model is more capable of handling morphological complexity among different rubber trees (with mIoU increased by 1.63 ​%, mAcc by 2.30 ​%, and mF1 by 0.50 ​%).

Despite the significant performance of the model in the experiments, there are still some limitations. Although we reduce the model's demand for computational resources by grouping the Transformer, in practical applications, TM-WSNet still requires high computational performance of the hardware, which limits its practical application on resource-constrained devices. Second, our model relies on a hybrid feature extraction module, which may suffer from insufficient feature extraction when the point cloud data is too sparse or has serious noise interference, thus affecting the segmentation effect. These shortcomings remind us to make further improvements in the future, and we will reduce the computational complexity by introducing more lightweight modules in the model architecture, such as sparse convolution, quantisation techniques or knowledge distillation. Moreover, developing adapted lightweight versions of the model for resource-constrained devices while keeping the core performance intact. Finally, stronger denoising mechanisms and adaptive feature extraction modules are designed and combined with data enhancement techniques to generate more sparse or highly noisy point cloud data to further extend the adaptability of the model.

## Author contributions

Lele Yan: Methodology, Writing-Original draft preparation, data acquisition. Guoxiong Zhou: Validation, Project administration, Funding acquisition. Minyin Yan: Supervision. Xiangjun Wang: Model guidance.

## Availability of supporting data

Data will be made available on https://github.com/YYYkHu/RubberTreeDataset.

## Declaration of competing interest

The authors declare that they have no known competing financial interests or personal relationships that could have appeared to influence the work reported in this paper.
